# A review of beyond citations for books: integrating library holdings and altmetrics in the impact evaluation of scholarly books and textbooks

**DOI:** 10.3389/frma.2026.1779778

**Published:** 2026-04-14

**Authors:** Ashraf Maleki

**Affiliations:** Department of Social Research, University of Turku, Turku, Finland

**Keywords:** altmetrics, book impact assessment, educational impact, library holdings, research evaluation, scholarly books

## Abstract

As scholarly books remain vital outputs in the humanities, social sciences, and education, new approaches are required to capture their diverse impacts. This study explores the multidimensional evaluation of academic books and textbooks by integrating citation metrics, alternative indicators, and (dis)aggregated library holdings data. Drawing on three large-scale empirical datasets encompassing over 119,000 Scopus-indexed book titles across 26 disciplines, the interactions and divergences among library print holdings (LPH), library electronic holdings (LEH), total library holdings (TLH), and other non-traditional metrics were examined in relation to their capacity to reflect scholarly and educational impacts. Library holdings data are among the most widely available book metrics (covering 97% of titles); however, aggregating print and electronic holdings into a single TLH metric has been shown to obscure important differences. Print holdings, though declining in average count over time, exhibit more stable, cumulative characteristics that statistically align with formal citation patterns, whereas electronic holdings reveal uneven acquisitions and inflated volumes that reduce the predictive strength of TLH. Print holdings outperform electronic holdings in modeling scholarly and educational impacts across most fields, except platforms such as Mendeley and Goodreads, which are better aligned with electronic availability. In a related investigation of textbooks, educational relevance is assessed through Open Syllabus Project rankings and compared with citation-based indicators (Scopus citations and book-to-book citation-based PageRank and HITS rankings), Goodreads user metrics, and WorldCat edition counts. Disciplinary differences emerge as prominent predictors of uptake across metrics. Goodreads ratings provide the strongest predictions in the humanities, WorldCat editions in the social sciences and medicine, and authority scores in citation networks in law and political science, improving the predictability of educational influence. Assessment of scholarly books benefits from acknowledging format-in-use and multidimensional usage patterns. As books often operate across scholarly, educational, and social contexts, their assessments should reflect blended purposes and audiences. Although a book's stated audience and intent may suggest a dominant context, substantial variation exists across titles. This provides a conceptual synthesis to integrate findings from large-scale quantitative studies on library holdings, citations, and altmetrics (alternative metrics) to present a more robust approach for assessing the multidimensional impact of academic books.

## 1 Introduction

Scholarly books and textbooks tend to hold longer-lasting influence compared to scholarly journal articles (Chi and Glänzel, 2024) within academia (Zhu et al., 2020) and in society, particularly in the humanities and social sciences (Tang, 2008; Pajić et al., 2019). Unlike journal articles, which are evaluated almost exclusively through citations, books inhabit a broader evaluative space that lasts longer and is influenced by multiple contexts and audiences (Crossick, 2018). Their reach spans the scholarly, educational, and social spheres, and the balance of these roles differs considerably across individual titles, which might be because particular audiences are the main target groups. Traditional citation analysis often does not fully reflect the impact of a book, and this enhances the importance of altmetrics (alternative metrics), which are non-citation indicators captured from online social media and external sources such as Goodreads and other books.

Although books are increasingly being published in a variety of formats, from letter-typed to audio and visual, the most commonly used forms, especially in libraries, are still known as print and ebook formats. Owing to significant differences in prices, libraries might acquire them for their collection based on different criteria. The library holding counts, which are the number of libraries providing loan access to book titles, might indicate early signs of book impact. However, library holding counts may be interpreted as an impact placeholder rather than a direct measure of realized influence, since library acquisition of books is faster and occurs before any other usage or impact (Maleki, 2022a). Acquisition by libraries does not necessarily imply scholarly use, citation, or educational adoption. However, print holdings are typically more expensive than ebooks and are associated with institutional demand assessments, collection development criteria, and anticipated relevance within specific academic or social communities (Romano, 2016). In this sense, they may reflect expectations of value rather than a confirmed impact. By ensuring access and long-term availability, library holdings establish infrastructural conditions that relate to readership, citation, educational or pedagogical use, and measurable engagement. Where available, loan or circulation statistics provide a more direct indication of realized usage (Romano, 2016), which is rarely accessible at scale for bibliometric research across print and e-books (Haugh, 2016). Holdings, therefore, represent a standardized and internationally comparable proxy for potential access, including forms of consultation and invisible readership that leave no trace of citation or altmetric systems. While holdings should not be equated with demonstrated influence, they may precede, enable, or condition the emergence of other measurable indicators in the broader context of scholarly communication. This makes examining its association with other impact indicators highly relevant and a convincing case for examining the potential of library holdings in book impact assessment, as signals drawn through this metric might enhance timely book impact assessment.

This study should be understood as a conceptual synthesis grounded in previously published large-scale empirical studies. Its purpose is to reinterpret and contextualize the responsible assessment of scholarly books across multiple metrics, publishing formats, disciplines, and temporal aspects of books. Rather than surveying the entire literature on book metrics, this study synthesizes format-specific, disciplinary, and temporal findings in order to highlight how different indicators relate to distinct dimensions of book impact. This synthesis draws on studies that investigated the use of multiple metrics that capture the uptake of books in relation to library holdings in print and electronic formats to capture the diverse forms of book impact in relation to their public supply through libraries.

The aim is to present how these measures interact, how the publishing format of books influences the relevance, predictability, and interpretability of various impact metrics, and what the implications of these different measures are in book impact assessment. Its purpose is to integrate the library-holding format, book impact indicators, and disciplinary and temporal findings into a unified study for evaluating scholarly books. While no new primary data are introduced, the synthesis aims to provide a structured reinterpretation of existing empirical results to clarify how publishing format, metric choice, disciplinary context, and time jointly shape responsible book impact assessments.

The added value of this conceptual synthesis lies in bringing together findings that were previously presented separately and demonstrating that the evaluation of scholarly books is simultaneously shaped by publishing format, metric choice, disciplinary variation, and temporal dynamics. From this synthesis, a format-based and normalized method emerges, in which print and electronic holdings, citations, educational indicators, and online engagement metrics are interpreted as complementary and non-equivalent signals of book influence.

## 2 Background

Scholarly books have long served as the foundation for research dissemination and education in the humanities, social sciences, and other professional disciplines. Unlike journal articles, which are routinely measured through citation-based indicators, books operate across multiple contexts, scholarly, educational, and social, each of which influences their reception and perceived value in distinct ways. Since the introduction of library holdings (also known as *Libcitations*) as an evaluative measure (White et al., 2009; Torres-Salinas and Moed, 2009), research has increasingly examined their capacity to indicate book impact. The WorldCat database, maintained by the Online Computer Library Center (OCLC), provides extensive data from over 15,000 libraries worldwide, representing a mixture of academic, public, and special institutions. The main strengths of this metric are as follows:

1. Broad coverage: Holdings are available for the vast majority of scholarly books (Maleki, 2022b).
2. Acknowledging multi-format accessibility: OCLC provides libraries' print and electronic holding counts separately, which facilitates an understanding of the acquired format and potential costs.
3. Fast in reflecting demand: Books, especially print holdings, are often acquired based on real demand, which relies on time and budgets. This enhances their inherent value as assessment metrics. As library acquisition facilitates reach and usage, it often signals other types of impacts.

However, transformations in academic library collection policies, particularly the expansion of electronic book acquisition, have complicated the interpretation of holdings data. While library print holdings (LPH) present deliberate, often permanent, acquisitions reflecting actual demand, library electronic holdings (LEH) are typically inflated through bundled publisher packages or subscription access, which may not signify actual demand, usage, or relevance. Prior studies (Romano, 2016; Wells and Sallenbach, 2015) have shown that many academic libraries now allocate the majority of their budgets to electronic resources, raising the question of whether total holdings (TLH, the combined count of print and electronic) overstate true scholarly engagement.

Empirical investigations have confirmed that electronic holdings exceed print holdings by a wide margin, often by a factor of seven, and that their ratio continues to grow for newer titles (Maleki, 2022b). Simultaneously, average print holdings are declining, mirroring budget shifts and readership behavior, rather than diminishing scholarly value. The increasing accessibility of the electronic format reduces its statistical value in assessment.

This synthesis brings together three major studies that have addressed these issues. The first two studies (Maleki, 2022a,b) examined the relationship between print, electronic, and total library holdings and multiple citation and altmetric indicators across 26 academic fields. The third study (Maleki et al., 2024) extends the investigation to the educational domain, testing how citation-based and non-citation indicators, including Goodreads engagement and book edition counts in WorldCat, predict the Open Syllabus Project (OSP) rankings of textbooks.

## 3 Method

Across the three studies, data were compiled for 119,794 Scopus-indexed book titles published between 1980 and 2018, representing 26 fields spanning the humanities, social sciences, and STEM fields. The initial dataset consisted of all Scopus-indexed books available in 2018 (*n* = 168,866), which was narrowed to 119,794 unique titles with ISBN/E-ISBN and subsequently to 110,603 titles with the OCLC Library of Congress classifications (Dataset 1).

Dataset 2 included 36,493 titles across six major subject categories (Anthropology, Arts, Business and Economics, Law, Medicine, and Political Science). For this subset, first-edition publication years were extracted from WorldCat to address inconsistencies caused by multiple republications and revised editions. Educational indicators (Open Syllabus Project data) were matched separately, resulting in 1,869 Scopus-indexed books with syllabus mentions.

### 3.1 Data sources and linking procedures

Each book title was linked across multiple data sources using ISBN/E-ISBN as the primary identifier and title-author matching as a fallback when necessary. Data were drawn from:

1. Scopus: citation counts and bibliographic metadata, including references.
2. OCLC WorldCat (classify.oclc.org; worldcat.org): library holdings and edition data.
3. Google Books: book-to-book citation counts.
4. Altmetric.com: Twitter/X, Facebook, blogs, news, Wikipedia mentions, and Mendeley readers.
5. Goodreads: user engagement indicators.
6. Open Syllabus Project (OSP): syllabus mentions.

Library print (LPH), electronic (LEH), and total holdings (TLH) were retrieved via OCLC API (January 2020) using ISBN-based primary searches and automated title-author fallback searches. TLH represents the sum of print and electronic holdings, as provided in the OCLC records. Google Books citations were extracted via API, following the method described by Kousha and Thelwall (2015). Altmetric indicators were collected via the Altmetric.com API (January 2020). Goodreads engagement data were retrieved via API (October 2018) using ISBN and title-author matching. Syllabus mentions were collected directly from the Open Syllabus Project (March–April 2020) using title and author matching, with manual verification where necessary. Book-to-book citations were collected via an author's customized application that matched the title and author name of syllabi that mentioned books in the entire Scopus dataset across all references using a Levenshtein similarity algorithm, followed by manual control of a set of references citing 80 books, resulting in 98% accuracy.

### 3.2 Variables

This study incorporated the following variables:

1. Library Holdings: Print (LPH), Electronic (LEH), and Total (TLH).
2. Citations: Scopus citations and Google Books citations.
3. Book-related Engagement Indicators: Goodreads users, ratings, and text reviews. Goodreads provides user-generated engagement data via its official API (active until December 2020). Prior research suggests that Goodreads engagement may reflect cultural, educational, and sometimes scholarly resonance of books, particularly in SSH fields (Zuccala et al., 2015; Kousha et al., 2017).
4. Altmetrics: Mendeley readers; mentions on Twitter/X, Facebook, blogs, news outlets, and Wikipedia. These indicators capture broader visibility and dissemination beyond formal citations (Hawkins, 2018; Taylor, 2020; Nan et al., 2020; Yang et al., 2021). Mendeley readership was analyzed alongside other altmetrics because it reflects academic saving behavior and may signal early scholarly attention.
5. Educational Indicators: The Open Syllabus Project mentions and WorldCat-indexed edition counts.
6. Network Measures (Textbooks): PageRank, HITS authority, and hub scores derived from book-to-book citation networks.

### 3.3 Analyses

All metrics were normalized using logarithmic transformations to address skewness and zero inflation, which are common in citation and altmetric data (Thelwall, 2017). Correspondingly, correlation and ordinary least squares regression analyses were conducted across disciplines and publication periods. Analyses were performed on near-population-level datasets comprising all Scopus-indexed books that met the inclusion criteria within the defined timeframe. Because the study did not rely on sampling but analyzed the full available corpus within the database scope, inferential statistics aimed at estimating broader population parameters were not central to the analytical design. Emphasis was therefore placed on the effect sizes, correlation magnitudes, and comparative explanatory power (adjusted *R*^2^). Where statistical significance is reported, it should be interpreted descriptively, rather than using a classical sampling study design.

## 4 Findings

### 4.1 Availability and coverage

Library holdings data demonstrated the highest coverage rate of all metrics, exceeding 97% across the 26 subject fields in the dataset, making them the most universally available indicator for scholarly books (Maleki, 2022b). By contrast, coverage for Scopus citations, which means a mix of citations from journal articles and Scopus-indexed scholarly books, ranged between 55% and 70%, Goodreads reviews averaged 25%, and Mendeley readership rarely surpassed 40%. This high availability positions holdings as an early and consistent signal of dissemination, measurable relatively sooner after publication than other metrics.

### 4.2 Print and electronic holdings in context

Although both print and electronic library holdings seem to be available for over 97% of books published in any given year shortly after publication, there are significant differences in their frequency of availability. Although print holdings still outnumber electronic holdings for older books (before 2003) by a substantial margin (mean ratio ≈ 7: 1), the gap is widening over time. Despite this numerical dominance, print holdings correlate moderately with research and educational indicators, whereas electronic holdings exhibit inconsistent acquisition patterns and significantly weaker statistical relationships with impact indicators (Maleki, 2022a,b).

The regression analysis showed that using disaggregated holding counts by print and electronic format counts separately in evaluations improves the impact prediction results. The results of the predictions are enhanced using print data as the primary baseline for long-term scholarly value and electronic data for accessibility analysis. Slight disciplinary differences may have influenced this prediction. However, as reading habits have changed, publishing habits have also changed. For instance, medicine, engineering, and mathematics are fields where electronic publishing and reading are more ingrained, and thus, the impact prediction patterns are linked with higher eholdings, Mendeley readership, and online Goodreads engagements (Maleki, 2022b).

**Figure 1:**
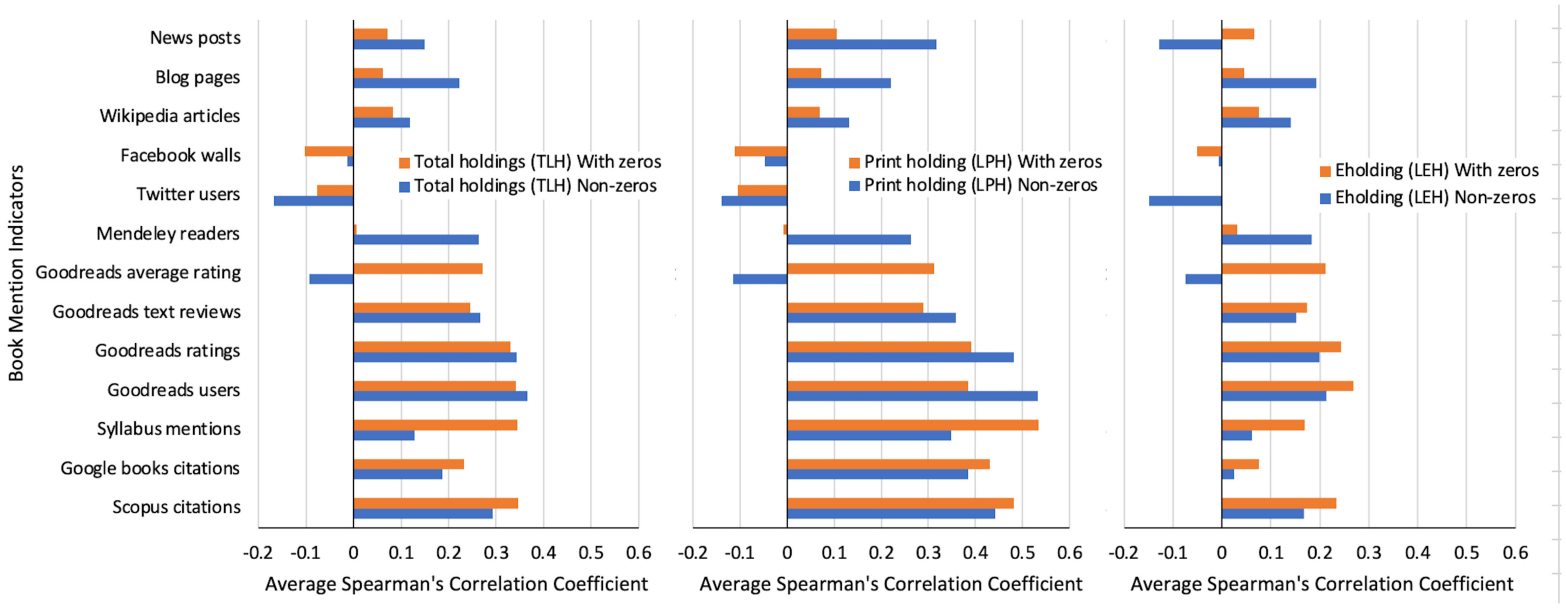
Effect of publishing format on book impact indicators shown by average median correlation coefficients across 26 fields, except for syllabus mentions of six fields (adopted from tables in Maleki, 2022b).

Figure 1 shows the average Spearman's correlation coefficients between pairs of scientometric indicators on the y-axis and the library holding counts across publishing formats in two situations: when zero metric counts are included and when they are excluded. LPH bars are consistently the highest for citations and syllabus mentions. LEH bars are the highest only for Goodreads. TLH bars are intermediate but weaker than the LPH bars. Publishing format fundamentally alters what “impact” library holdings capture and is strongest when print holdings are used independently.

### 4.3 Correlations and predictive power

Across all fields, total holdings displayed only weak correlations with other metrics, confirming previous findings (White et al., 2009; Torres-Salinas et al., 2021). However, when disaggregated, print holdings exhibited moderate relationships with both citations and educational metrics, potentially clarifying some prior studies that observed contradictory findings when comparing US libraries with holding counts in other countries without accounting for book format. There seems to be a tendency in US-based libraries to record significantly higher levels of eholding counts compared to libraries in other countries, which is deduced only from a comparison with the results of a previous distinct study of the holding counts of history books between American Research Libraries (ARL) and non-ARL libraries. This indicated extremely weak correlations between holdings and Goodreads counts for ARL holdings but moderate correlation for non-ARL holdings in the top 75th percentile of metric counts (Zuccala et al., 2015). In our study of the field of history, we observed that disaggregating print and eholdings seems to indicate comparable differences, since all correlation coefficients at different percentiles and total data between print holdings and Goodreads metrics and Scopus citations for history books were at the medium level, where eholdings show weak correlations (Maleki, 2022a). This is analogous to the differences between ARL and non-ARL Holdings' results from Zuccala et al. (2015), although making direct assumptions is not possible because of the unavailability of data on the disaggregation of holding types across US and non-US libraries.

In the same study, print holdings correlated most strongly with Google Books citations, which shows that book-to-book citations frequently make the best predictor in regression models (14 out of 26 fields), especially in fields where books are a prominent output type. In contrast, Goodreads user counts, despite being weak overall, were the main predictors of electronic and total holdings (15 fields), reflecting the stronger alignment of e-holdings with online popularity (Maleki, 2022a). In sum, the inclusion of electronic holdings in total counts was shown to dilute the predictive value of library holding metrics. Print holdings, in contrast, are consistently moderately correlated with scholarly and educational use, underscoring their theoretical and statistical validity for impact assessment (Maleki, 2022a).

**Figure 2:**
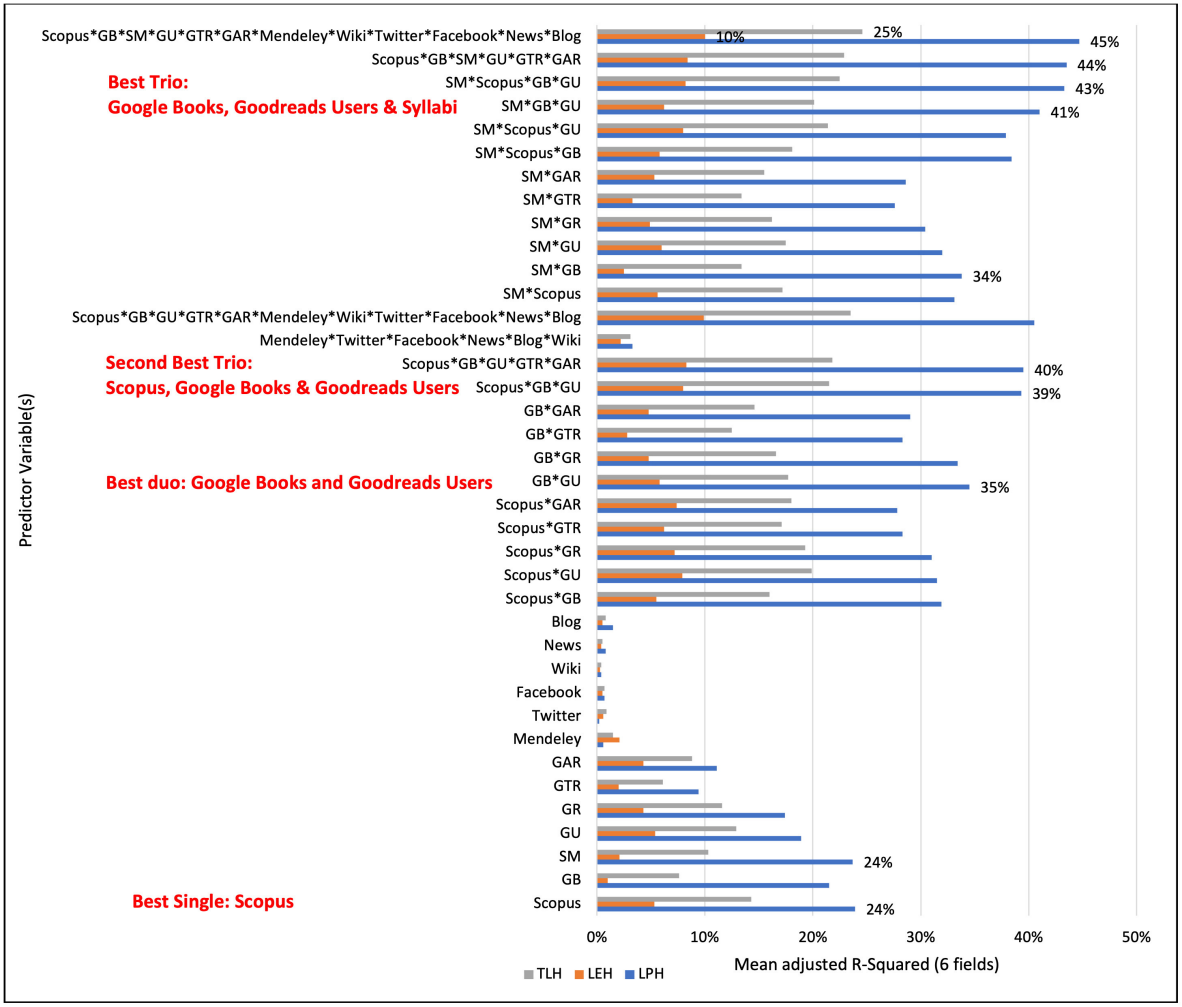
Variability of library holding model outputs across different metric specifications is shown by mean adjusted *R*^2^ values from ordinary least squares regressions. Library print holdings (LPH), electronic holdings (LEH), and total holdings (TLH) were modeled as dependent variables, with citation, altmetric, and educational indicators entered either individually or in combination as independent variables. Values represent averages across all 26 fields in Dataset 1 and across six fields (Dataset 2) when syllabus mentions (SM) were included in the model (adopted from Maleki, 2022b). GB, Google Books; SM, Syllabus Mentions; GU, Goodreads Users; GR, Goodreads Rating; GTR, Goodreads Text Reviews; GAR, Goodreads Average Ratings.

Figure 2 shows that the best single variables to account for print holding were Scopus Citations and Syllabi Mentions captured from the Open Syllabi Project (both approximately 24%). The combined regression with two variables explaining the variance in print holdings was Google Books Citations and Goodreads User Counts (~35%). This was only slightly improved by adding a third variable, usually mentioned by Syllabi (41%), which explained the variance better than Scopus (39%). Including all variables only accounted for 45% of print holdings, but this was by far the best prediction on the impact of books beyond eholdings (merely 10%) and the compromised count of total holdings (25%). This shows that book impact varies strongly with metric choice, and no indicator is universally superior, whereas there is a significant overlap and differences in the variance explained by different metrics. The highest complementarity was between Google Books and Syllabi mentions, which together explained 34% of print holdings and 22% to 24% when used alone.

#### 4.3.1 Temporal and disciplinary patterns

**Figure 3:**
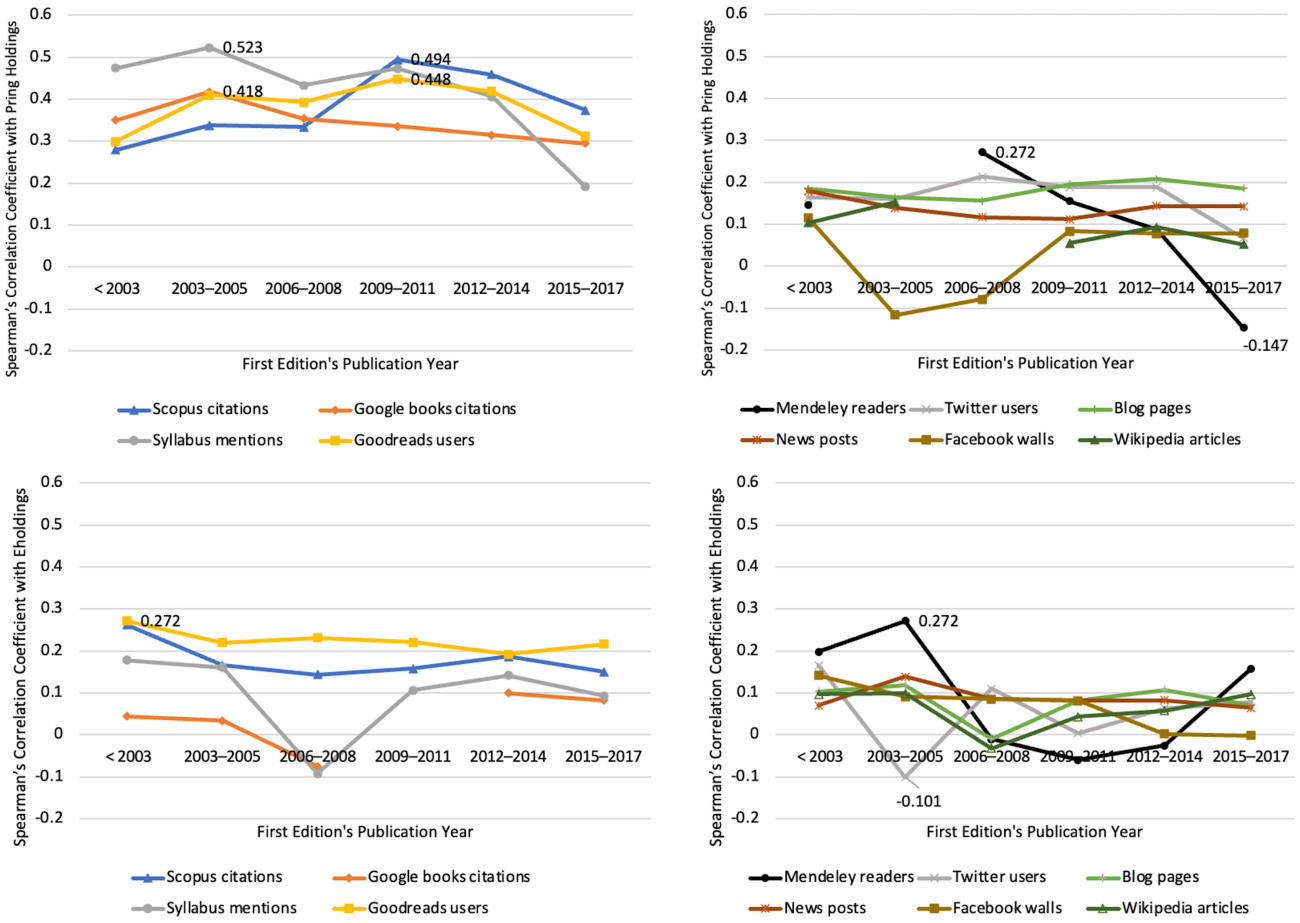
Temporal dynamics of book impact indicators by Spearman's median correlation coefficient between library holding formats and other impact indicators (adopted from Maleki, 2022b).

The holdings-based impact patterns vary substantially across fields. Humanities and social science disciplines, particularly philosophy, history, and political science, exhibited the highest levels of both print holdings and educational uptake, whereas medicine and engineering showed the strongest growth in electronic holdings. Temporal analyses, in Figure 3, revealed that the correlation between print holdings and other metrics peaked for books published around 2003–2005, corresponding to the era before large-scale ebook acquisitions (Maleki, 2022a). From 2010 onwards, the correlations weakened, suggesting that the surge in e-holdings introduced statistical noise rather than a meaningful signal. This aligns with library acquisition policies that increasingly favor electronic bundles over targeted print purchases (Romano, 2016).

**Figure 4:**
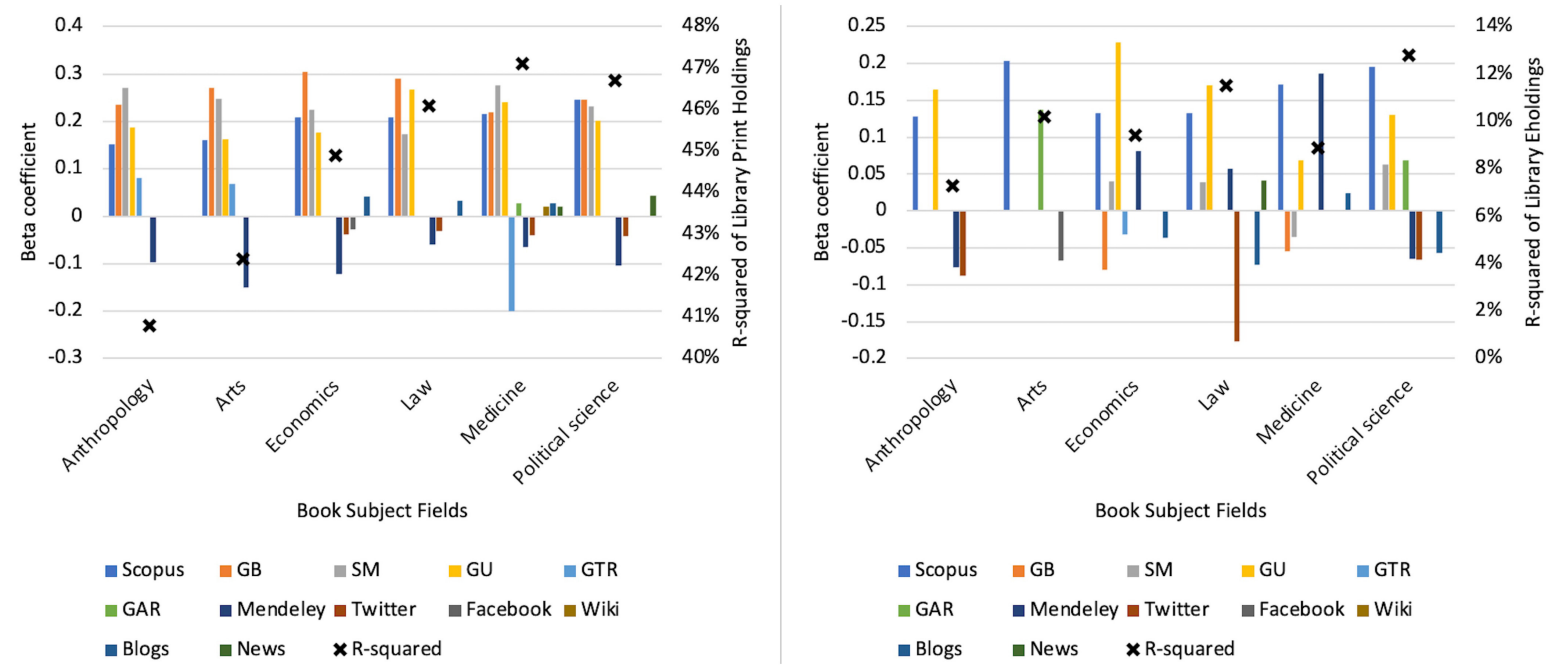
Disciplinary variation in book impact signals based on mean statistically significant linear regression standardized coefficients (β) in the six fields. In the **left** figure, the dependent metric is library print holdings, and in the **right** figure, it is eholdings. GB: Google Books; SM: Syllabus Mentions; GU: Goodreads Users; GTR: Goodreads Text Reviews; GAR: Goodreads Average Ratings.

The dissemination of print holdings is moderately correlated with academic and educational use and readers' online engagement over time. Social media continuously indicates both weak positive and negative but statistically significant correlations with any form of library holding (Figure 4). Mendeley readership tends to give more negative signals at the beginning, before becoming positive, but is still weakly correlated with holdings later on.

Unlike most indicators, which have relatively similar performance, Mendeley readers that are weakly and often negatively connected with print holdings are positively but still weakly connected to eholdings, playing a significant role in impact assessment in medicine, engineering, and mathematics. Unlike other fields, medicine benefits from a higher alignment of Mendeley with eholdings, but overall, it does not lead to a better statistical prediction of impact than print holdings, suggesting that even in fields with a high presence of eholdings, impact assessment is not significantly enhanced.

### 4.4 Educational impact: predicting syllabus adoption

Another study (Maleki et al., 2024) expands the analysis to examine the educational impact of the inclusion of books in syllabi and course descriptions using a dataset of 1,869 academic textbooks indexed in Scopus. Educational relevance was determined using the Open Syllabus Project rank, which represents the frequency of textbook adoption in university curricula. Both citation-based and non-citation indicators were investigated, and regression models for different disciplines achieved predictive accuracies ranging from 32% (technology) to 68% (philosophy, psychology, and religion). The key findings were the following:

- WorldCat edition counts were the strongest predictors in social sciences, medicine, and technology, which denotes the updated use of books, and publisher re-issuance (reprinting and editing) characterizes educational value.
- Goodreads ratings predicted educational rank and scholarly usage most effectively in the humanities, suggesting an overlap between them.
- Citation network authority scores (HITS) predicted syllabus adoption in law and political science more than in any other field, demonstrating that intellectual centrality contributes to educational influence.

These findings indicate that no single metric adequately represents educational impact; rather, combinations of citation, edition, and engagement metrics enhance our understanding of the statistical requirements of books for more accurate impact predictions.

## 5 Limitations and discussion

A main limitation of this study concerns the nature of the present contribution as a conceptual synthesis of previously published empirical studies. The study mainly focused on highlighting the relative importance of book metrics, and depending on the scope, design, data sources, and metric choices of the original studies, no new primary dataset was constructed specifically for the synthesis itself. Accordingly, the conclusions drawn here should be understood as an integrative reinterpretation of those studies rather than as an independent empirical study based on newly assembled data.

Books are inherently multicontextual objects. Their impact cannot be confined to a single evaluative dimension, sometimes even when it has one target group, as they simultaneously operate within the scholarly, educational, and social spheres in various proportions. Books designed primarily for academic research may achieve secondary educational value, whereas textbooks may accrue citations through theoretical originality or disciplinary authority. This multidimensionality is particularly characteristic of the humanities and social sciences, where monographs remain central in the scholarly context and enjoy a longer citation life cycle than journal articles (Pajić et al., 2019; Chi and Glänzel, 2024). Consequently, evaluation frameworks must acknowledge that books balance these contexts in varying proportions across disciplines and target audiences. This position aligns with broader arguments that monographs require evaluation approaches that are distinct from journal-centric models (Crossick, 2018). This study, in line with other studies, demonstrates that holdings, citations, and altmetrics capture different aspects of book impact.

- Library print holdings: institutional recognition and scholarly value (White et al., 2009; Torres-Salinas and Moed, 2009; Torres-Salinas et al., 2021).
- Citations: integration within academic knowledge networks (Kousha et al., 2011; Zhu et al., 2020).
- Altmetrics (Goodreads, Mendeley, and social media mentions): public, societal, early scholarly attention, and educational value (Hawkins, 2018; Thelwall, 2021; Nan et al., 2020; Yang et al., 2021).
- WorldCat editions and OSP ranks: pedagogical uptake and teaching impact (Kousha and Thelwall, 2016; Maleki et al., 2024).

Thus, interpreting book impact requires attention to both publishing format and context, with the understanding that each metric reflects a different type of audience relationship.

Format-Specific Assessment Implications: Libraries with various publishing formats seem to reflect scholarly access. Caution must be taken when aggregating print and electronic holdings into a single total holding metric value because it obscures significant differences in acquisition logic, distribution, and meaning. The rapid expansion of bundled ebook acquisition models in academic libraries (Romano, 2016; Wells and Sallenbach, 2015) explains why electronic holdings may inflate total counts without proportionate evidence of scholarly demand. Although library holdings, particularly print holdings, demonstrate consistent associations with scholarly and educational indicators, total holding counts should not be interpreted as direct measures of quality or realized impact. Aggregated total holdings may inflate perceived influence due to bundled ebook acquisitions. Institutional collection policies vary geographically and economically, potentially introducing structural bias. Using holdings as ranking tools requires contextual normalization. Holdings are best interpreted as infrastructural or demand-based signals that require compartmentalization of impact values based on acquisition formats and complementary indicators for responsible applications.

Metric-Related Implications: Citations, holdings, and altmetrics each capture partial perspectives of influence. Hybrid models that combine these indicators outperform single-metric approaches and can accommodate disciplinary variations better. The print-holding variance was statistically explained much better than the other two holding variables through the inclusion of all other impact variables. This finding supports previous evidence that citation and altmetric indicators often reveal complementary rather than substitutive impact dimensions (Kousha et al., 2017; Yang et al., 2021). At the same time, caution is warranted in interpreting altmetric signals as a direct societal impact, given known methodological limitations and susceptibility to noise (Thelwall, 2021).

The conceptual distinction between the role of different library book-holding statistics is presented by regression analyses. By modeling print holdings (LPH), electronic holdings (LEH), and total holdings (TLH) separately against combinations of citation-based, altmetric, and educational indicators, differences in the explanation of impact signals specific to print and ebook acquisition became visible. Print holdings consistently showed stronger and more stable associations with scholarly and educational metrics, suggesting an alignment with established academic demand and institutional valuation. In contrast, electronic holdings were more strongly associated with online engagement indicators and exhibited weaker or less consistent relationships with citation-based measures. Total holdings, when modeled as an aggregated variable, demonstrated reduced explanatory coherence, indicating that the inclusion of electronic acquisitions may dilute the interpretive clarity of print-based demand signals. These regression results do not imply a causal direction between metrics; however, they reinforce the view that publishing format mediates how library holdings relate to other observable dimensions of book influence. In this sense, the predictive patterns observed in the models empirically reflect the infrastructural and demand-based logic underlying the different holding formats.

Field-Level Implications: Disciplinary context shapes which indicators meaningfully capture book impact. Because publication, teaching, and reading cultures vary widely across fields, impact models remain discipline-specific. Indicators such as Goodreads engagement are more meaningful in the humanities, whereas WorldCat editions better represent textbook persistence in the social sciences (Maleki et al., 2024). The citation authority in a network of book citations strongly predicts Law and Political Science's educational book impact, whereas STEM fields have lower predictability overall. These disciplinary differences are consistent with the established publication and citation patterns in SSH, whereas monographs hold sustained relevance and citation windows are longer (Pajić et al., 2019; Chi and Glänzel, 2024). Furthermore, prior work has shown that alternative indicators may align more closely with communication practices in the humanities than in STEM contexts (Zuccala et al., 2015; Taylor, 2020).

Temporal Implications: Holdings accumulate rapidly after publication and stabilize, whereas citations and syllabi adoption develop gradually. This temporal distinction aligns with broader findings on citation obsolescence, where books often exhibit slower aging patterns than journal articles in the SSH field (Chi and Glänzel, 2024). The dissemination of print holdings was moderately positively correlated with academic and educational use and readers' online engagement over time. Social media continuously indicates both positive and negative weak but statistically significant correlations with any form of library holding (Figure 4). Mendeley readership tends to give more negative signals at the beginning before becoming positively weakly correlated with holdings, suggesting differences between early attention signals and long-term institutional validation.

Overall, the synthesis of these studies supports the idea that book evaluation benefits from moving beyond citation analysis to library holdings, book-to-book citations (Kousha and Thelwall, 2015), educational usage (Kousha and Thelwall, 2016), and social media platforms. Library holdings, especially in print, should be retained as a central indicator, interpreted alongside both scholarly and educational metrics, because of their alignment with more meaningful usage, needs, and expectations. The inclusion of non-citation indicators such as Goodreads and syllabus data provides a more holistic view of how books engage distinct audiences across scholarly and societal domains.

These findings also contribute to the ongoing reform debates in responsible research assessment by demonstrating that journal-derived evaluative logic cannot be directly transferred to book-oriented disciplines. Formatted aggregation, metric cultures, and a lack of field normalization risk systematic bias against humanities and social sciences scholarships. Therefore, a responsible framework for book evaluation must integrate multiple indicators, distinguish library-acquired publishing formats, and interpret metrics within disciplinary and temporal contexts to enhance the recognition of the wide spectrum of book influence.

## 6 Conclusion

This study summarizes these three studies to establish an empirical foundation for advancing the evaluation of scholarly books and textbooks. Beyond integrating the findings of the three original studies, this study contributes to the conceptual and methodological position of book evaluation. Conceptually, it proposes that scholarly books should be assessed as multicontextual objects whose influence unfolds across scholarly, educational, and societal domains. Methodologically, it recommends separating print and electronic holdings, avoiding the uncritical use of aggregated total holdings, and utilizing citation, educational, and alternative indicators after normalizing for discipline and time. The highlighted evidence also demonstrates the following:

- Total holdings (TLH) should be used cautiously, because aggregation conceals meaningful distinctions.
- Print holdings remain the most valid and interpretable measure of academic and educational impact.
- Electronic holdings, while abundant, often reflect access models rather than genuine demand and perform statistically weak associations, indicating an impact.
- Scholarly indicators, such as formal journal article citations to books and Google Books, offer significant academic perspectives, help to identify the knowledge-based authority of books, and are predictable from library holdings in a large-scale analysis.
- Educational and social indicators, such as syllabus mentions, Goodreads ratings, and WorldCat editions, offer indispensable complementary perspectives in line with library print holdings.

The added value of this synthesis of several population-scale studies of book metrics lies in integrating the empirical findings across these studies in terms of library-holding format and disciplinary, temporal, and attention platform-specific dimensions from a coherent, responsible evaluation perspective. This helps to advance methodological and theoretical clarity of use of book-related metrics beyond the distinct studies it draws upon by demonstrating that altmetrics do not apply to the majority of books, unlike library holdings; however, availability or lack of availability of books in libraries presents a chance of visibility, attention, and even real-world impact in the context of education or cultural and scientific change. It also suggested that, unlike journal articles, altmetric indicators, such as Mendeley readership and social media attention, might not align with actual readership impact, whereas Goodreads might provide more powerful signals. This study also gave a ranked value of impact with regard to scholarly books' library holdings and statistical alignment with citation and non-citation metrics. Various platforms demonstrate a powerful message on how attention metrics should be used cautiously for books, while enhancing the value of libraries' book-holding statistics that preserve the integrity of book assessment. Books are multidimensional entities situated across intersecting scholarly, educational, and social contexts. Therefore, their impact evaluation must move beyond singular indicators toward integrated hybrid frameworks that respect this plurality. By focusing on multiple indicators of holdings, citations, and altmetrics, the evaluation of books can achieve fairness and inclusivity in representing the diverse roles that books continue to play in the advancement of knowledge and education.
